# 0D Nanofillers in EPDM-Based Elastomeric Ablatives: A Review of Thermo-Ablative Performance and Char Formation

**DOI:** 10.3390/polym18030405

**Published:** 2026-02-04

**Authors:** Mohammed Meiirbekov, Marat Nurguzhin, Marat Janikeyev, Zhannat Kadyrov, Mukhammed Sadykov, Assem Kuandyk, Nurmakhan Yesbolov, Nurlybek Spandiyar, Meiir Nurzhanov, Sunkar Orazbek

**Affiliations:** 1 Joint-Stock Company “National Center of Space Research and Technology”, Almaty 050010, Kazakhstan; meiirbekov.m@spaceres.kz (M.M.); nurguzhin.m@spaceres.kz (M.N.); m.janikeyev@spaceres.kz (M.J.); zh.kadyrov@spaceres.kz (Z.K.); m.sadykov@spaceres.kz (M.S.); a.kuandyk@spaceres.kz (A.K.); n.yesbolov@spaceres.kz (N.Y.); s.nurlybek@spaceres.kz (N.S.); 2 Faculty of Mechanics and Mathematics, Al-Farabi Kazakh National University, Almaty 050040, Kazakhstan; 3 Department of Automation and Control, Institute of Telecommunications and Automation, Almaty University of Power Engineering and Telecommunications Named After Gumarbek Daukeyev, Almaty 050013, Kazakhstan; 4 School of Materials Science and Green Technologies, Kazakh-British Technical University, Almaty 050005, Kazakhstan; 5 Mining and Metallurgical Institute Named After O.A. Baikonurov, Kazakh National Research Technical University Named After K.I. Satbayev, Almaty 050043, Kazakhstan

**Keywords:** EPDM, ablation, thermo-ablative composites, 0D nanofillers, carbon black, metal oxide nanoparticles, char layer formation, SRM

## Abstract

EPDM is widely used as the polymer matrix for solid rocket motor (SRM) internal thermal protection because of its low density, chemical inertness, and ability to form carbonaceous residue. Practical performance is frequently limited by weak char integrity and barrier properties, char oxidation, mechanical stripping in gas-dynamic flow, and by the poor comparability of published results due to non-uniform test conditions and reporting. This review systematizes studies on 0D nanofillers in EPDM ablatives and harmonizes the key metrics, including linear and mass ablation rates (LAR, MAR), back-face temperature (T_back_), and solid residue yield. The major 0D additives-nSiO_2_, nTiO_2_, nZnO, and carbon black (CB) are compared, and their dominant mechanisms are summarized: degradation-layer structuring, reduced gas permeability, thermo-oxidative stabilization, and effects on vulcanization. Several studies report larger improvements for hybrid systems, where CB enhances char cohesion and retention, while oxide nanoparticles improve barrier performance and resistance to oxidation. Finally, an application-oriented selection matrix is proposed that accounts for thermal protection efficiency, processability, agglomeration limits, and density penalties to support EPDM coating design and improve comparability.

## 1. Introduction

Thermal protection systems of solid rocket motors (SRMs) operate under conditions of intense heat and mass transfer, where the temperature of combustion products reaches ~2900 °C and the heat flux to the inner surface of the chamber can attain ~4.5 MW·m^−2^. These regimes impose a combined thermal, chemical, and mechanical load on the thermal protection material [1,2,3]. Experimental studies have shown that, under such conditions, the linear ablative rate (LAR) of the inner chamber surface can reach ~0.22 mm·s^−1^. This parameter is commonly used in the literature as a quantitative measure of material degradation intensity [4,5,6].

**Figure 1:**
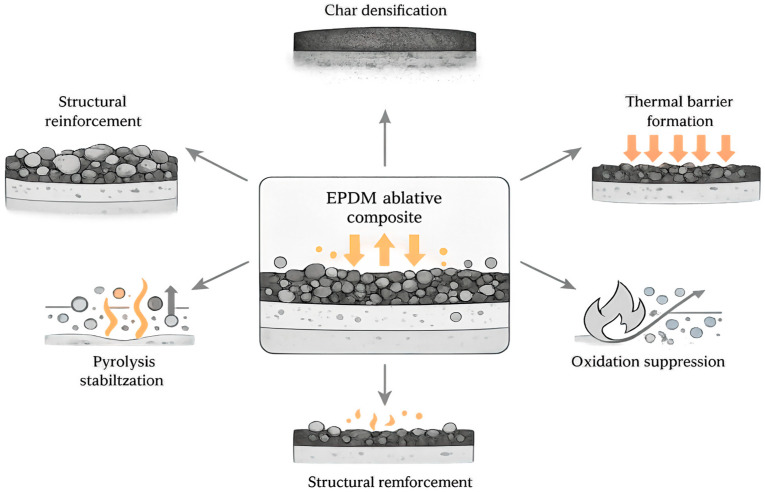
Schematic overview of coupled degradation and protection mechanisms in EPDM ablative composites.

Under these conditions, ablative thermal insulation materials operate through a combination of thermal protection mechanisms, including heat absorption and the formation of a char layer [7]. Heat absorption is associated with endothermic decomposition of the polymer matrix and the transport of pyrolysis gases, which affect heat and mass transfer in the near-surface region [8,9]. The formation of a char layer, in turn, reduces the thermal load on the subsurface layers and modifies the interaction between the material and the surrounding gas phase [7,10]. For polymer-based ablative systems, the structure and stability of the formed char layer are generally regarded as key factors governing ablation resistance, since this layer largely controls heat and mass transfer and the degradation behavior of the material [7,8,10]. These coupled degradation and protection mechanisms are summarized schematically in Figure 1.

Historically, thermal protection materials have included carbon-based composites, including a carbon–carbon composite, ceramic materials and coatings, as well as polymer-based ablative systems [11,12]. Recent comparative reviews of modern SRM thermal protection systems highlight that a thermal protection system’s performance is inseparable from manufacturing technology, since routes such as sintering, infiltration, polymer-forming, and additive manufacturing directly constrain achievable architectures and reliability under extreme thermo-mechanical loading [13]. In the context of SRMs, polymer thermal protection coatings, particularly those based on ethylene propylene diene rubber (EPDM), have become widely adopted due to the combination of their low density of ~0.85 g·cm^−3^ and their ability to form a strong interfacial bond with the motor casing, including through co-curing [14,15]. This broader dependence on the process and architecture is also evident in the structural composite systems relevant to aerospace hardware, where the filament winding angle strongly shifts the strength balance in carbon-fiber-reinforced polymer tubes [16]. Similarly, glass and aramid epoxy composites demonstrate architecture-dependent dielectric permittivity and radio transparency, reinforcing that manufacturing route and reinforcement choice often define functional performance envelopes [17]. Among elastomeric matrices used for flexible ablative materials, nitrile rubbers, EPDM, polyphosphazene elastomers, and silicone rubbers are the most commonly employed [18]. Considering the overall balance of properties, EPDM is often regarded as a promising matrix for elastomeric thermal protection coatings for SRMs, as it is characterized by low density, chemical resistance, and the ability to form a protective carbonaceous layer during ablation [19]. Collectively, these features enable the development of EPDM-based composite materials that combine mechanical reliability, ablation resistance, and thermal insulation efficiency [14].

Despite the substantial body of research on EPDM-based systems, a number of limitations remain that complicate the transfer of the literature results into engineering practice. The char formed in EPDM composite materials is often characterized by insufficient integrity, elevated porosity, and fragmentation, which leads to increased gas permeability and can promote oxidative degradation at high temperatures. An additional limitation is the high sensitivity of filler effects to test conditions and formulation details, resulting in discrepancies in reported performance, even for apparently similar compositions [14,20]. A further critical issue is the lack of a unified format for reporting performance characteristics. LAR, MAR, T_back_, and solid residue yield are determined under different test regimes and specimen geometries, which hinders direct comparisons of data.

In this context, one of the most widely discussed approaches to improving the thermal protection efficiency of EPDM is the use of nanofillers [21]. Compared with micro- and macro-fillers, nanoparticles exhibit a high specific surface area and pronounced interfacial activity, which enables effective modification of the composite structure at low loadings [20,22]. For EPDM ablators, it is particularly important that nanophases can simultaneously influence vulcanization kinetics, interfacial adhesion, and the formation of the char layer [20,23,24].

From a review perspective, nanophases should be distinguished according to dimensionality and morphology, since, for high-aspect-ratio one-dimensional and two-dimensional fillers, particle orientation may lead to property anisotropy and the increased sensitivity of the material to deformation [25,26].

The zero-dimensional (0D) nanoadditives considered for EPDM ablative systems include oxide nanoparticles and CB [20,22,23]. In particular, silicon dioxide nanoparticles (nSiO_2_) can promote the formation of a more compact char layer, which is commonly associated with a reduction in T_back_ and an improvement in thermal protection efficiency. However, under elevated heat fluxes, the char layer may lose its protective function due to erosion, which highlights the requirement for sufficient thermomechanical stability of this layer [27,28].

Titanium dioxide nanoparticles (nTiO_2_) and zinc oxide nanoparticles (nZnO) are considered functional inorganic additives that influence charring behavior, solid residue yield, and the properties of the protective layer. For nTiO_2_, a potentially nonlinear dependence of the effect on filler content has been reported, whereas nZnO, in addition to its role in vulcanization, may enhance condensed phase mechanisms in flame-retardant systems [29,30,31].

Carbon-based zero-dimensional phases (CB) act as effective reinforcing fillers. In EPDM ablative systems, the strength and integrity of the char layer largely determine its resistance to mechanical erosion by the combustion gas flow [14,32]. In this context, different zero-dimensional nanoadditives affect different rate-limiting stages of EPDM degradation, and the greatest effect is expected for hybrid architectures.

In light of the above, the objective of this review is to systematize and compare published results on the influence of zero-dimensional nanofillers on the thermoablative properties of EPDM-based composite materials. The work emphasizes the unification of terminology and performance metrics, as well as the analysis of mechanisms linking the type of nanophase to the formation of the char layer. In addition, technological limitations related to the dispersion and agglomeration of nanofillers, as well as their influence on vulcanization and mixture rheology, are discussed [20,33,34]. At the implementation level, reproducibility is often limited not only by formulation, but also by process equipment and controlled material delivery, as illustrated by engineering improvements in automated graded spreading units and controlled crack-opening milling technologies [35,36]. The outcome of this review is the development of an application-oriented framework for selecting zero-dimensional nanoadditives according to the engineering task, enabling the correlation of degradation scenarios, protective mechanisms, and expected performance effects, thereby improving the reproducibility of EPDM thermal protection coating design.

## 2. Nanofillers for EPDM

**Figure 2:**
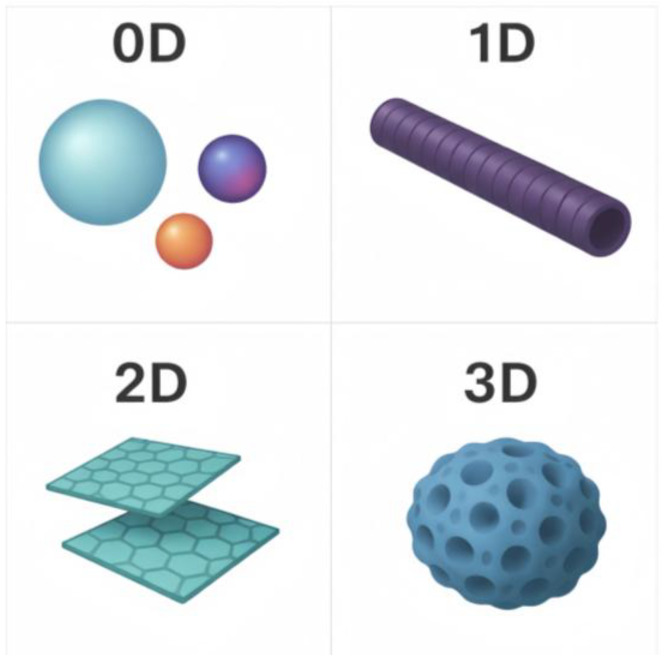
Classification of nanofillers by dimensionality (0D, 1D, 2D, and 3D) and representative mechanisms of action.

Nanofillers are defined as fillers that possess at least one characteristic dimension in the range of 1–100 nm. A reduction in particle size is generally accompanied by an increase in specific surface area and an expansion of the interfacial contact area with the polymer matrix [37,38]. The contribution of the nanophase is determined not only by the chemical nature of the particles, but also by their morphology, since morphology, particularly specific surface area, can influence dispersion quality and the intensity of interfacial interactions with the matrix [22,39]. Nanomaterials are also commonly classified according to dimensionality as zero-dimensional, one-dimensional, two-dimensional, and three-dimensional, with representative examples reported for each category [40,41]. A schematic classification and representative mechanisms are summarized in Figure 2.

In EPDM-based thermal protection systems, different morphological types of nanomaterials generally activate different mechanisms. One-dimensional nanophases such as carbon nanotubes and nanofibers can promote the formation of extended rigid elements, including framework-like or micro-network structures, within the char layer and, when sufficient connectivity is achieved, increase the compactness and mechanical strength of the carbonaceous residue [14,20,22]. Two-dimensional nanoplatelets, such as layered silicates and graphene-like structures in polymer nanocomposites, are commonly associated with improved gas barrier properties due to an increase in the effective diffusion path length, often described as a tortuous diffusion path [42,43,44]. For EPDM ablators, the potential relevance of this effect is related to restricting the oxidizer’s access to the pyrolysis zone. However, the gas barrier’s contribution should be interpreted with caution and considered together with its influence on the structure and mechanical integrity of the char layer [45,46,47]. Three-dimensional nanostructures such as aerogels and porous frameworks are characterized by a three-dimensional nanoporous architecture and, as a result of nanoscale pores, may provide very low effective thermal conductivity [28,48,49].

Anisotropic one-dimensional and two-dimensional nanofillers are capable of orienting during processing or deformation, and such an orientation can significantly affect the contribution of the filler to mechanical properties and reinforcement of the composite material. When spherical or particle like additives are used, the formation of more isotropic composite materials is often observed due to the relatively free spatial distribution of particles within the matrix [50]. For ablative materials, it is essential that thermal protection efficiency is determined not only by the properties of the initial polymer matrix, but also by the characteristics of the pyrolysis layer and the char layer formed under thermal exposure, including their contribution to thermal insulation and the mechanical stability of the overall system [14].

In particular, parameters such as the density, degree of cracking, adhesion to the underlying substrate, and oxidizer permeability are commonly considered in the literature as a combined set of factors that can influence the thermal behavior of ablative materials. This includes their resistance to heat flux, gas diffusion processes, and the characteristics of structured pyrolysis product formation [10,14,51].

In this context, 0D nanoparticles are regarded as potential fillers capable of affecting the morphology of pyrolysis layer and the microstructure of the char layer. As noted in several studies, this influence may be associated with increased density, reduced defect concentration, and improved mechanical properties of the protective char layer under heat flux exposure [10,51].

In EPDM-based thermal protection systems, it is more appropriate to relate the comparatively processing-robust contribution of 0D fillers not to an inevitable isotropy of the resulting structure, but to the fact that isotropic-in-shape particles depend less on processing-induced orientation and tend to express their effects primarily through dispersion quality and the magnitude of accessible interfacial surface area, including the interphase and bound rubber. This is consistent with general concepts on the role of the specific surface area and interfacial interactions in polymer nanocomposites [52,53,54]. By contrast, for anisotropic 1D/2D fillers, pronounced reinforcing and barrier effects more often require favorable alignment, a sufficient degree of exfoliation, and/or the formation of a percolated network; therefore, the contribution of such fillers is more strongly dependent on shear history and processing conditions, which can lead to direction-dependent transport, including permeability and heat transfer, as well as increased sensitivity to local heterogeneities in orientation and connectivity [55,56,57,58].

**Figure 3:**
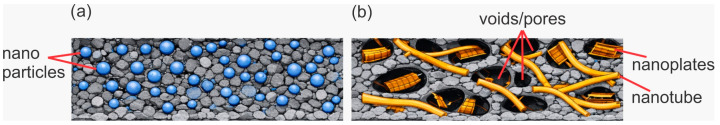
Schematic illustration of the influence of filler dimensionality on composite microstructure and pore formation: (**a**) composite with dispersed 0D nanoparticles forming a relatively uniform particulate-filled structure; (**b**) composite with 1D nanotubes and 2D nanoplates, leading to the formation of voids and interconnected pore regions due to geometric constraints.

For ablative EPDM systems, the key factor remains the formation and integrity of the pyrolysis residue and the char layer. Char morphology, including pore size and pore connectivity, is associated with transport within the porous skeleton, oxidizer diffusion, and, consequently, the progression of thermochemical processes in the protective residue. For EPDM insulators, characteristic compact and porous char-layer structures have been reported, highlighting the role of porosity and structural heterogeneity [59]. Introducing well-dispersed additives has been reported to densify the residue and/or shift the char toward a finer-pore morphology in EPDM-based systems [28]. Such microstructural refinement is consistent with reduced permeability and restricted oxidizer supply, which can improve erosion resistance and strengthen the overall thermal protection effect under oxidative flow [60], as schematically illustrated in Figure 3. At the same time, char cracking during pyrolysis, associated with thermally induced shrinkage stresses, creates additional transport pathways and reduces the barrier properties of the protective residue, in line with general models of cracking during pyrolysis [61]. Overall, the reported superiority of 0D fillers is more appropriately interpreted as a context-dependent outcome governed by EPDM grade, formulation, test conditions, and char-formation mechanisms, rather than as a universal advantage over 1D/2D fillers [59,62].

At severe ablation temperatures, oxidation of carbonaceous char can be viewed as a competition between oxygen delivery to reactive carbon surfaces and Arrhenius-type surface reaction kinetics on the accessible carbon area. As connected porosity, permeability, and crack opening evolve during exposure, the apparent oxidation response can shift between transport-limited oxygen supply and reaction-limited surface oxidation, so materials with comparable carbon chemistry can exhibit different apparent recession across exposure modes with different boundary-layer transport, pressure, shear, and oxidizer availability [63,64,65]. This regime-based view clarifies the use of the term antioxidant in EPDM-based ablatives by separating radical-scavenging concepts commonly discussed for thermo-oxidative aging from transport-controlled oxidation during ablation. Fillers may act as radical scavengers under aging conditions, while under ablative exposure, they can reduce apparent oxidation losses by promoting a denser and mechanically more stable residue that restricts access of oxidizing gases to reactive carbon surfaces [14,66,67].

Accordingly, additives that densify the residue, reduce open connected pathways, and increase tortuosity can lower the effective oxygen flux through the char, while additives that strengthen the char skeleton can reduce shrinkage-driven cracking, fragmentation, and peel-off events that continually expose fresh material and renew reactive surface area. In addition, the growth of an inorganic or ceramic-like surface component can form a partial barrier that shields the carbonaceous phase and further shifts behavior toward diffusion-impeded oxidation [51,68,69].

For EPDM-derived chars, pore connectivity and char density govern gas and oxidizer transport, and silica-derived surface enrichment can contribute to a more compact outer layer that limits oxidizer access. Under controlled dispersion, carbon nanofillers can increase the continuity of the carbonaceous scaffold and support char retention, while silica and other oxides are widely used to densify the surface region and reduce apparent oxidation losses through transport limitation. In this review, it is interpreted as coupled oxygen diffusion and surface reactions that depend on the evolving pore structure and local density rather than as a regime-independent intrinsic parameter [20,59,70].

Oxide inorganic additives and nanofillers, including nSiO_2_, nTiO_2_, and nZnO, are considered in studies of EPDM composite materials as tools for inorganic modification [71,72]. For nSiO_2_-containing formulations, effects on the yield and morphology of the char residue are reported, including the formation of a more effective degradation layer structure, which is interpreted as a factor governing thermal protection performance [28]. Studies on EPDM ablators and thermal protection composites have shown that changes in composition, including ablative additives and inorganic fillers, can alter LAR and MAR as well as the thermal response of the back face. When interpreting such data, it is important to consider that the formed carbonaceous residue, namely the char layer, performs a barrier function by resisting erosive action of the high temperature flow. In addition, the size and connectivity of pores within the char layer affect heat and mass transfer and the release of pyrolysis gases. Studies have further reported that a reduction in cracking and changes in the porous structure of the char layer accompany improvements in its integrity during high-temperature testing [14,62].

In a number of studies on EPDM-based composite materials, oxide fillers are considered as a means of modifying the thermal and performance characteristics [28,73,74]. For EPDM, the use of modified fumed silica belonging to the SiO_2_ type, as well as TiO_2_, in some formulations in combination with other additives, has been reported alongside an analysis of their influence on thermal stability and composite properties [71]. It has also been shown that replacement of conventional ZnO with nZnO may be accompanied by a delay in thermal degradation according to TGA data and by changes in degradation kinetic parameters [72]. For SiO_2_-containing EPDM systems, it is reported that fumed silica and silica aerogel affect the morphology of the pyrolysis residue through the formation of a thinner and denser char framework with smaller and more uniformly distributed pores. This effect may be accompanied by a reduction in the thermal conductivity of the char skeleton [28]. Studies on EPDM ablators have further shown that modification of material architecture can reduce T_back_ in oxygen acetylene tests [14] and be accompanied by changes in LAR and MAR, depending on composition and filler type [62].

In EPDM-based ablative and thermal protection systems, the formation of a carbonized char layer is regarded as a key factor determining performance, with particular emphasis on the thermal stability and integrity of the char [14]. CB is cited as one of the most widely used carbon fillers for EPDM composite materials [22]. It has been shown that the porous char layer formed during ablation can perform a barrier function by resisting the erosive action of the high temperature flow. The size and connectivity of pores within the char layer can influence the interaction between heat flux and the surface, as well as the release of pyrolysis gases, reflecting the role of protective layer morphology in the heat and mass transfer processes in the near-surface region [20]. It is also reported that modification of composite formulation may lead to the formation of a denser and more compact protective layer structure, which is associated with reduced diffusion of heat and oxygen into the material and enhanced thermo-oxidative stability [51]. Reductions in cracking and changes in the porous structure of the char layer are typically accompanied by improved integrity during high-temperature testing [10].

In the literature on EPDM nanocomposites, CB is mentioned among organic nanofillers used to modify the properties of EPDM [22]. Studies on EPDM composite materials for thermal insulation and ablative protection have shown that filler selection affects thermal and ablative characteristics [62]. It is emphasized that the use of high-filler loadings may lead to increased density and thermal conductivity. Therefore, material architecture and design strategies are explored that allow for the improvement of the thermal protection response without an excessive increase in filler content [14].

**Figure 4:**
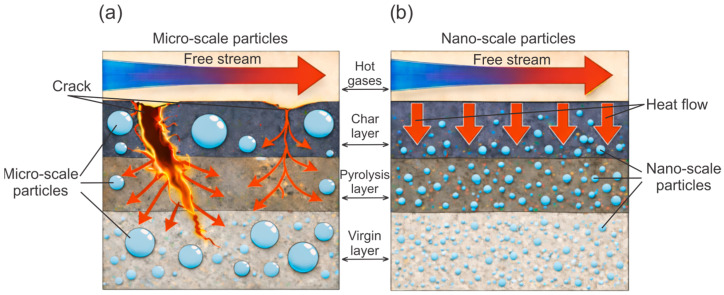
Effect of particle size on EPDM pyrolysis and char layers: (**a**) micro-scale particles with cracked, less coherent char; (**b**) 0D nanoparticles with denser, more uniform layers and reduced cracking.

Comparisons of quantitative ablation characteristics and thermal response of EPDM-based systems reported in different studies may depend to a certain extent on features of the experimental methodologies employed. Such features include differences in the type of thermal exposure, the level of applied heat flux, specimen geometry, and the approaches used to evaluate ablation rate. Consequently, analyses and discussions of the literature data require careful consideration of the specific conditions under which the corresponding tests were conducted. In this context, the influence of filler particle size on the morphology and integrity of the pyrolysis and char layers is schematically illustrated in Figure 4.

Nanoparticles can agglomerate during elastomer compounding because high surface energy and interparticle attractions tend to promote aggregation that reduces exposed surface area. Therefore, the realized dispersion state is often governed by processing and deagglomeration history rather than by nominal primary particle metrics alone. Consequently, nominal specific surface area does not necessarily equal the effective accessible specific surface area in rubber because aggregation and incomplete deagglomeration reduce interfacial area and can attenuate the interphase even when high-specific-surface-area primary particles are used [75,76].

For SiO_2_-type 0D fillers, silanol-rich surfaces can promote strong filler interactions via hydrogen bonding, which is a widely recognized contributor to poor silica microdispersion in nonpolar rubbers unless interfacial engineering and mixing strategies suppress filler contacts [77,78]. In filled rubbers, the interphase is frequently discussed via bound rubber, and bound rubber measurements are commonly used as an indicator of the rubber filler interactions associated with reinforcement [79,80]. Under controlled dispersion, a broader effective contact area can increase the fraction of constrained chains and make the interphase more spatially uniform, whereas persistent agglomerates localize interfacial contributions and reduce the potential property gains from nanofillers [76,79].

In EPDM thermal protection and ablative systems, this dispersion issue is particularly relevant because char performance is often morphology controlled. Char layer studies explicitly report compact porous structuring and discuss how pore architecture and connectivity govern internal transport through the residue [59]. Therefore, comparisons between 0D versus 1D and 2D fillers should separate geometry-linked effects from dispersion-controlled effects such as effective accessible surface area and agglomerate breakup, and, for 1D and 2D fillers, additionally account for orientation, exfoliation state, and network connectivity [81,82].

In EPDM with octaphenylsilsesquioxane, the authors showed poor dispersion. Aggregates of about 10–15 µm are reported, and octaphenylsilsesquioxane tends to form block-like domains around 160–200 nm. In line with this, low resistance to long-term hot-flame erosion is reported, and at 500 °C the burn-through time is about 1300 s. For EPDM with fumed silica in the same study, homogeneous dispersion in the EPDM matrix is reported. For the EPDM/Si composite, backside-temperature data are provided. At 500 °C, the backside remains around 242–250 °C at 100 s, and at 1000 °C, it reaches about 388 °C at 800 s. The char is shown as a thicker scaffold with larger porosity, with an average pore diameter of about 108 nm and a porosity of about 76%. For EPDM with silica aerogel, more uniform distribution is reported and inclusions are barely visible. Most particles are in the 3–25 nm range. In backside tests, the EPDM/Gel composite gives 176–186 °C at 100 s at 500 °C, and at 1000 °C, the backside reaches about 433 °C at 800 s. The char pores show an average diameter of about 43 nm and porosity of about 68% with a denser structure, although the char surface after 1000 °C can be strongly damaged [28].

### 2.1. Zinc Oxide Nanoparticles (nZnO)

Zinc oxide ZnO is among the most widely used functional additives in elastomer formulations and has traditionally been employed as a component of vulcanization systems, influencing crosslinking kinetics and network structure [30,83]. In EPDM composite materials intended for thermal protection and operation under intense thermal exposure, ZnO should be considered not as a thermal insulation filler, but rather as a functional additive that regulates interfacial and degradation-related processes and that is capable of modifying vulcanization parameters and thermal degradation kinetics [23,72].

**Figure 5:**
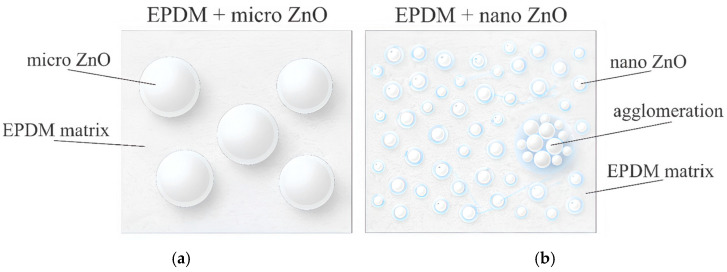
Micro and nZnO in EPDM: (**a**) micro ZnO with limited interfacial contact; (**b**) nZnO with increased interfacial contact and agglomeration tendency.

From the viewpoint of basic physicochemical properties, ZnO is a dense oxide with a stable crystalline structure of the wurtzite type with hexagonal symmetry [84,85]. Crystalline ZnO is characterized by a relatively high thermal conductivity on the order of ~50 W·m^−1^·K^−1^, which can exhibit pronounced anisotropy at the single-crystal level, with representative values of ~44 W·m^−1^·K^−1^ along an in-plane (a-axis) direction and ~62 W·m^−1^·K^−1^ along the c-axis direction [86]. However, in practical EPDM formulations, the dominant factors are not the properties of ZnO’s single crystals, but rather its dispersion state, degree of aggregation, and the quality of interfacial contact with the elastomer matrix [22]. The transition from microscale ZnO to nZnO is accompanied by an increase in specific surface area from units of m^2^·g^−1^ to approximately 20–60 m^2^·g^−1^ and by an increase in the fraction of the interfacial region, which enhances particle–matrix interactions [87]. At the same time, the system’s sensitivity to dispersion conditions increases. When the optimal filler loading is exceeded or mixing efficiency is insufficient, the risk of agglomeration and the formation of structural heterogeneities rise [88,89]. The corresponding differences in interfacial contact and agglomeration tendency for micro-ZnO versus nZnO are schematically illustrated in Figure 5.

Experimental studies of EPDM composites show that the replacement of conventional ZnO with nZnO is accompanied by a delay in thermal degradation according to thermogravimetric analysis data (TGA) [72]. Kinetic analysis of TGA data using the Friedman and Kissinger methods indicates a substantial change in degradation kinetic parameters. The activation energy increases by 132–145%. The same study also shows that the use of nZnO in EPDM formulations can lead to a reduction in vulcanization time and an increase in crosslink density compared with systems containing conventional ZnO, indicating the particle-size-dependent influence of ZnO on the vulcanization network parameters [23].

ZnO plays an important role through its influence on vulcanization and therefore on the initial network structure, which largely determines crack resistance, mechanical integrity, and retention of the degradation layer under thermal exposure [72]. In sulfur vulcanization systems, ZnO is commonly regarded as a component that promotes the formation of active complexes with accelerators in the presence of co-activators, including fatty acids [30,90]. This is associated with accelerated vulcanization kinetics and the possibility of achieving higher crosslink density. In peroxide-based systems, the mechanisms differ; however, interfacial effects and the dispersion state of ZnO may also affect the kinetics and homogeneity of the resulting network structure [91,92].

From a practical perspective, for thermal protection applications, a key conclusion is that an optimized vulcanization network contributes to the preservation of mechanical integrity during heating and facilitates the formation of a stronger and more stable protective char layer [93,94].

**Figure 6:**
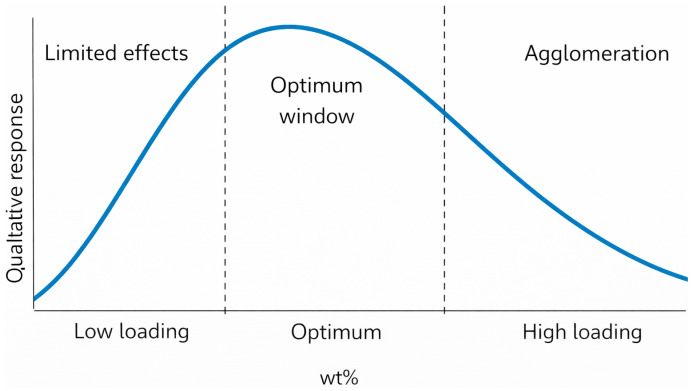
Qualitative schematic illustrating a generic dose-dependent response of EPDM-based ablative systems to oxide nanofiller loading, highlighting low-loading (limited effects), an optimum window, and high-loading (agglomeration-related deterioration) regimes.

The sensitivity of nZnO to dispersion quality is a critical factor that determines the morphology and performance characteristics of rubber composites. Self-aggregation of nanoparticles can disrupt distribution uniformity and be accompanied by deterioration of certain mechanical properties, whereas more uniform dispersion is generally associated with more stable material behavior [72,95]. From a processing perspective, this implies that the beneficial effects of nZnO are realized only under controlled dispersion conditions, while agglomeration can lead to the formation of local heterogeneous regions, including areas with increased crosslink density [89]. A qualitative illustration of nZnO in EPDM, emphasizing the dispersion and agglomeration effects, is summarized in Figure 6.

When analyzing heat transfer in polymer composite materials, the contributions of fillers and interfacial effects should be taken into account. ZnO is described as a thermally conductive inorganic component, while the agglomeration of nanoparticles can modify the effective thermal conductivity of the composite material, leading either to an increase of up to ~22% or to a decrease, depending on the degree of aggregation and the structural features of the system [96,97,98]. In elastomer formulations, ZnO simultaneously retains its primary role as a vulcanization activator. Under ablative conditions, the thermal protection response is realized through pyrolysis and ablation, including the oxidative removal of surface residue, spalling, and sublimation processes [30,99,100,101].

**Figure 7:**
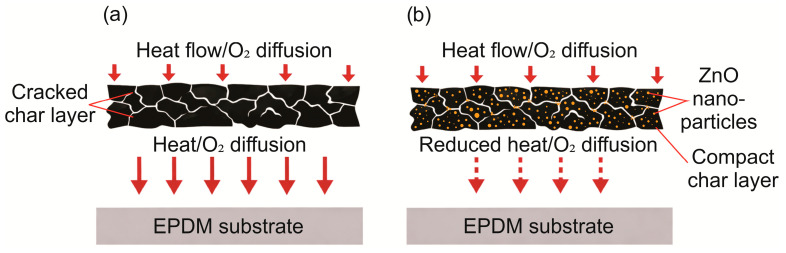
Conceptual effect of ZnO on char layer compactness and oxidizer diffusion in EPDM: (**a**) cracked char layer enabling enhanced heat and O_2_ diffusion to the EPDM substrate; (**b**) compact char layer containing nZnO with reduced heat and O_2_ diffusion.

The ablation resistance of EPDM-based composite materials is determined primarily by the structure and mechanical stability of the char layer. A compact and mechanically robust char layer can restrict oxygen diffusion and physicochemical erosion, thereby reducing the ablation rate, whereas increased porosity, fragmentation, and weakening of the char residue facilitate penetration of heat flux and accelerate surface degradation [14,20]. This conceptual role of ZnO in densifying the char layer and limiting oxidizer diffusion is illustrated in Figure 7.

The literature contains numerous reviews and experimental studies in which ZnO and nZnO are mainly considered as components of elastomer vulcanization systems. There are also studies on EPDM that address the influence of nZnO on mechanical properties and thermal degradation behavior. However, direct quantitative comparisons of the contribution of ZnO and nZnO to LAR and MAR under high-heat-flux conditions remain limited. In several EPDM studies employing oxygen acetylene torch testing, indicative values of LAR on the order of 10^−2^ mm·s^−1^ have been reported, depending on the formulation and test conditions [14,23,30,72,102].

Accordingly, the role of ZnO in the ablative behavior of EPDM is more appropriately interpreted, as indirectly supported through its influence on vulcanization network structure, thermal degradation kinetics, and char layer morphology, rather than as a guaranteed reduction in ablation losses. The most substantiated strategy appears to be the use of ZnO within hybrid systems, where the primary contribution to the formation of a ceramic-like and barrier layer is provided by other fillers, while ZnO performs a stabilizing function at optimized loadings.

### 2.2. Silicon Oxide Nanoparticles (nSiO_2_)

nSiO_2_-containing fillers introduced into EPDM can serve different purposes depending on the application. They can improve the mechanical properties and hydrogen barrier performance in sealing compositions, and they can enhance the thermal protection efficiency of EPDM-based composite materials under flame exposure. The latter effect is typically manifested by a reduction in T_back_ and the formation of a denser and more compact char layer in systems containing fumed silica or silica aerogel [28,103,104].

**Figure 8:**
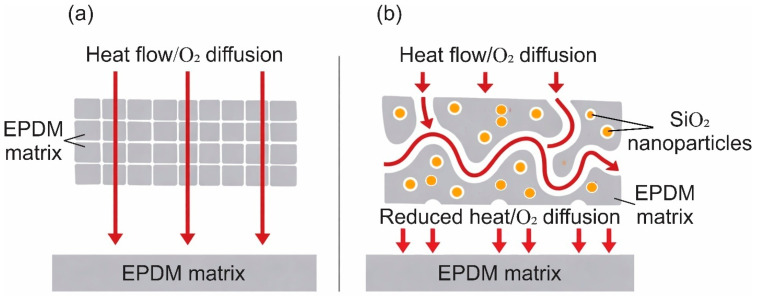
Conceptual effect of nSiO_2_ on heat and O_2_ diffusion in EPDM: (**a**) neat EPDM with direct heat and O_2_ diffusion paths; (**b**) EPDM with nSiO_2_ with tortuous paths and reduced heat and O_2_ diffusion.

nSiO_2_-containing fillers, commonly referred to as silica, used in both sealing and thermal protection system formulations based on EPDM, can substantially modify a material’s structure and properties. For EPDM-sealing compositions, an increase in silica content in the presence of a silane coupling agent is reported to enhance filler–rubber interactions and crosslink density. This is accompanied by increases in modulus and tensile strength together with a decrease in hydrogen permeability and hydrogen diffusivity. This diffusion barrier mechanism is schematically illustrated in Figure 8.

For EPDM-based thermal protection composite materials exposed to flame temperatures in the range of 500–1000 °C, the addition of fumed silica or silica aerogel is associated with increased char residue and the formation of a more compact char layer. This behavior is commonly correlated with a reduction in T_back_ [28,103,105].

For EPDM composites containing nSiO_2_-based fillers, including silica aerogel, some studies report, within a single investigation, both mechanical properties and the results of ablative or flame tests. These include LAR, T_back_ measured at 500–1000 °C, and characteristics of the char residue. Such sets of metrics are convenient for the quantitative comparison of EPDM formulations, provided that the test protocols are comparable [28,106].

nSiO_2_ is described in the literature as a filler and property modifier for EPDM composites, including systems intended for thermal insulation and thermal protection that incorporate nSiO_2_-containing additives [22,28]. For amorphous nSiO_2_, often referred to as silica glass, density values on the order of 2.20 g·cm^−3^ are reported in several studies [107,108]. The size characteristics of silica are governed by the degree of aggregation. Primary particles are on the nanometer scale, whereas aggregates and agglomerates can reach hundreds of nanometers or even micrometer dimensions [109,110]. For silica aerogels, specific surface area values in the range of 945–1283 m^2^·g^−1^ are reported, that is, exceeding 1000 m^2^·g^−1^ [111]. The surface chemistry of nSiO_2_ allows for the functionalization, for example, with siloxane groups, which is widely used to modify and study the interactions between silica surfaces and organic or inorganic polymers [112]. The addition of fumed silica can enhance thixotropic behavior and increase viscosity and yield stress in composite systems [14].

nSiO_2_ containing fillers are considered across several research directions related to EPDM-based materials [28,103,113]. In sealing compositions, it has been shown that increasing silica content reduces hydrogen permeability and hydrogen diffusivity [103]. In studies on EPDM-based thermal protection composite materials, fumed silica and silica aerogel are associated with changes in char layer structure and with a reduction in T_back_ during burn-through testing [28]. For porous nSiO_2_ fillers, particularly silica aerogel, a reinforcement mechanism based on the formation of clusters and a three-dimensional physical network within EPDM is discussed, with the magnitude of the effect depending on the specific surface area and porosity of the filler [14].

**Figure 9:**
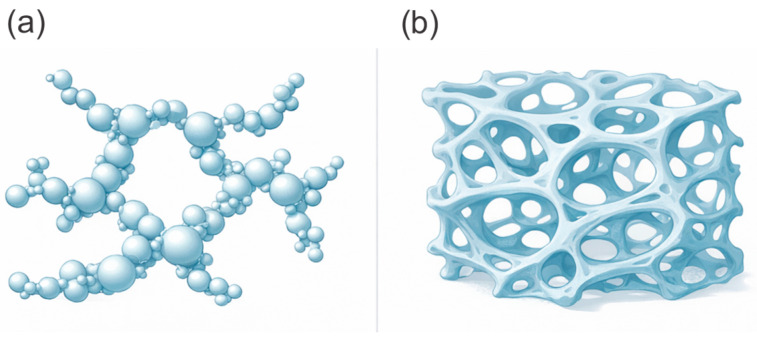
Forms of nSiO_2_ used in EPDM composites: (**a**) fumed SiO_2_ providing reinforcement and gas barrier effects; (**b**) SiO_2_ aerogel characterized by high porosity and ultralow thermal conductivity.

Review studies on silica aerogels emphasize their high porosity and very low thermal conductivity on the order of ~0.01 W·m^−1^·K^−1^, which explains the interest in these materials as thermal insulation components [114,115]. Comparative studies of EPDM composites containing fumed silica and silica aerogel demonstrate that these fillers differ in thermal conductivity and behavior under flame exposure [75]. In flame tests, T_back_ is commonly used as a performance metric, with representative values such as 388 °C after 800 s reported for a selected formulation within a given comparison. Within this comparative framework, the lower intrinsic thermal conductivity of the filler, as in the case of silica aerogel, did not necessarily translate into improved thermal protection metrics in thermal protection metrics. Instead, the yield of char residue and the structural characteristics of the formed char layer remained the dominant factors governing thermal protection behavior [28]. The two commonly used nSiO_2_ forms in EPDM composites and their expected functional differences are summarized schematically in Figure 9.

The mechanical properties of EPDM-based composite materials filled with silica change with increasing filler content. In the studied formulations, an increase in reinforcement effect and improved mechanical properties are observed with increasing silica loading expressed in parts per hundred rubbers (phr). Reviews of rubber nanocomposites emphasize that the mechanical response is also sensitive to the dispersion state of the filler and to polymer–filler interactions. During compounding, the formation of agglomerated structures and material heterogeneities may occur [89,103]

For EPDM filled with silica, increasing filler content may result in both an increase in modulus and strength and a deterioration of certain properties due to agglomeration. The rheological behavior of the mixture is also strongly dependent on filler content and dispersion quality [103]. From a processing perspective, this implies the need for careful control of mixture rheology and filler distribution uniformity at elevated loadings, particularly for nanoscale dispersed fractions. Relevant data, including the influence of silica on modulus, strength, and elongation, are summarized in Table 1.

Table 1 is used here to provide a compact formulation-level context for subsequent thermal-protection discussion. Wherever possible, the listed values correspond to within-study comparisons, because absolute tensile metrics are sensitive to EPDM grade, curing system, and processing history.

In EPDM-based thermal protection composite materials, the addition of fumed silica is associated in several studies with the formation of a denser and more stable char layer, which increases erosion resistance and reduces the likelihood of burn-through. In comparative studies conducted at 1000 °C, systems containing fumed silica show an increased yield of char residue and the formation of a compact and porous char layer. In contrast, for compositions containing silica aerogel, the lower intrinsic thermal conductivity of the filler does not always result in an improved thermal protection response unless accompanied by a corresponding modification of the char layer’s structure [28].

Overall, the datasets in Table 1 indicate that SiO_2_-filled EPDM commonly exhibits increased strength and hardness, while elongation at break can vary with dispersion quality and interfacial design. This room temperature mechanical context is relevant for ablative applications because the ability of the near-surface residue to remain cohesive under flow is often linked to the integrity of the evolving pyrolysis and char layers, so Table 1 should be treated as an indirect indicator of the pre-pyrolysis network state rather than a direct predictor of ablation. Variations in dispersion quality and cure state, including crosslink-density-controlled stiffness and extensibility, as well as the extent of a bound-rubber-type filler–rubber interphase, can be used as practical proxies of how uniform the EPDM network is before heating [80,121,122]. During thermal exposure, shrinkage during solid pyrolysis can localize stresses and promote cracking once local stresses exceed the cracking threshold of the degrading solid [61]. The resulting crack density and connected transport pathways can facilitate oxidizer ingress and internal transport through the residue, shifting oxidation susceptibility and the ability of the char to survive gas/particle erosion [5,59]. Accordingly, differences in crack density and connected porosity can translate into different oxidation/erosion losses and thus into different LAR, MAR, and T_back_ trends under otherwise comparable exposure [59].

Microstructural observations of EPDM-based insulators containing silica under oxygen acetylene ablation conditions, with variations in the content of multi-walled carbon nanotubes, reveal densification and a reduction in the characteristic pore size within the char layer. In these studies, this behavior is attributed to the carbothermal reduction of silica at high temperatures with the formation of silicon carbide in the ablation zone. For hybrid EPDM Kevlar nanocomposites, the addition of nSiO_2_ is accompanied by a reduction in thermal conductivity, while thermal stability is evaluated using TGA [20,119]. Comparable ablative parameters, including linear and mass degradation rates as well as indicators of thermal response, are summarized in Table 2. Because these metrics are highly test-dependent, meaningful comparisons should be interpreted primarily within identical test configurations and specimen geometries.

**Figure 10:**
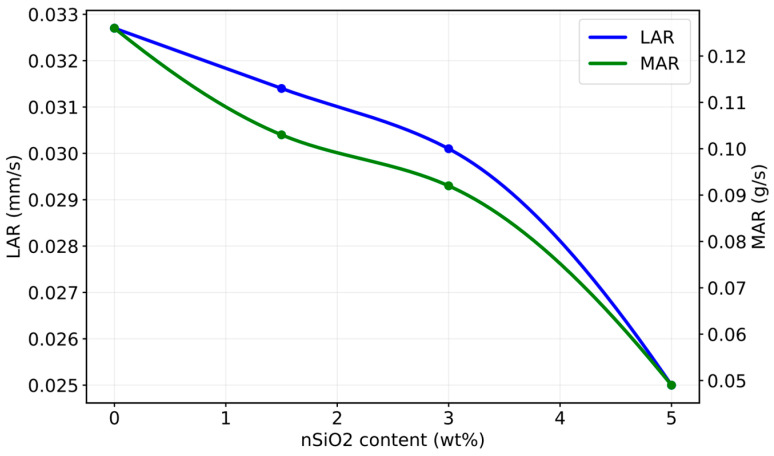
Effect of nSiO_2_ content on LAR and MAR under oxy acetylene torch testing for carbon-fiber phenolic bulk-molding compound.

As an additional illustration of how nanosilica can reduce ablation losses, the results reported by Mirzapour et al. for a carbon-fiber-reinforced phenolic bulk-molding compound containing nSiO_2_ at 0–5 wt% under oxy acetylene torch testing can be cited (Figure 10). With increasing nSiO_2_ content, a monotonic decrease is observed in both the LAR and the MAR [124].

Comparative studies of EPDM-based thermal protection composite materials containing fumed silica and silica aerogel show that behavior under flame exposure is closely related to the type of silica filler and to the characteristics of the formed char layer. For systems containing fumed silica, a more compact internal char layer is observed compared with EPDM gel-based composites, and the formed char layer can effectively protect the polymer matrix and prevent continuous flame induced ablation. Silica aerogels as a class are characterized by very high porosity, with reported values exceeding 90%, and very low thermal conductivity, for example, in the range of 0.012–0.024 W m^−1^ K^−1^. At 1000 °C, EPDM gel-based composites exhibit severe damage on the char layer surface [28,125].

Flame torch tests of EPDM-based composite materials conducted at 500 °C and 1000 °C show that changes in composition lead to differences in T_back_ values and in the surface condition of the char layer. Published data also demonstrate that the measured characteristics depend on the EPDM grade and the filler system employed [105,126,127,128,129,130]. Accordingly, meaningful comparisons are valid only for fixed-test regimes and well-defined specimen geometries [28,62].

Thermal analysis in air for EPDM-based composite materials containing fumed silica and silica aerogel reveals differences in char residue relative to neat EPDM. For the aerogel-containing formulation, the TGA temperature at 5% mass loss (T_5_%, temperature at 5% mass loss) is reported to decrease, which the authors attribute to the mass loss associated with residual low-boiling organic components in the silica aerogel and/or to the decomposition or oxidation of hydroxyl, methyl, and other organic groups present on the surface [28]. In the context of thermal protection, the char layer is regarded as a key factor governing ablative performance, since it can protect the polymer matrix and resist heat diffusion and physicochemical erosion by combustion products [14,28].

**Figure 11:**
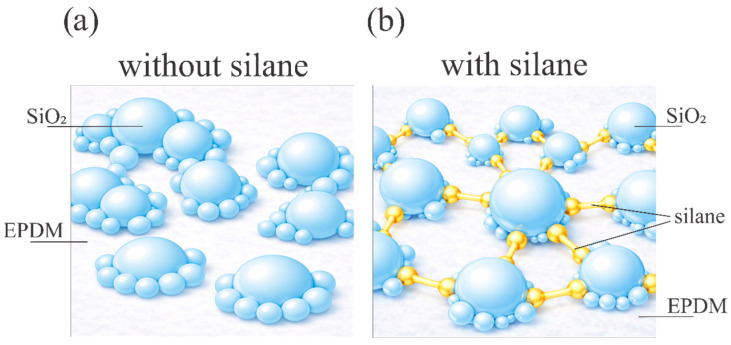
Effect of silane coupling on nSiO_2_ dispersion and interfacial bonding in EPDM composites: (**a**) without silane, showing nSiO_2_ agglomeration and weaker interfacial connectivity in the EPDM matrix; (**b**) with silane, showing improved nSiO_2_ dispersion and silane-mediated interfacial bridging in the EPDM matrix.

The limitations associated with the incorporation of nSiO_2_ into EPDM are often related to achieving uniform dispersion at elevated filler loadings. With increasing nSiO_2_ content, enhanced agglomeration and restricted chain mobility are reported. In addition, for EPDM nSiO_2_ systems, a reduction in effective crosslink density has been shown, which is attributed to the influence of nSiO_2_ on components of the crosslinking system, for example, partial consumption of the initiator and the interfacial role of the coupling agent. This effect can potentially influence the mechanical properties [22,120]. In sealing compositions, increasing silica loading, expressed in parts phr, is typically accompanied by enhanced reinforcement and improved mechanical performance. However, the quantitative balance between strength, deformability, and rheology remains formulation-dependent and requires consideration of the specific mixing and vulcanization system employed [28,89,103]. The role of silane coupling in improving nSiO_2_ dispersion and interfacial bonding is schematically illustrated in Figure 11.

Overall, nSiO_2_ in EPDM is among the more consistently reported fillers from the perspective of thermal protection behavior. It simultaneously enhances mechanical stability and promotes the formation of a more stable char layer, which is reflected in the ablation-related parameters and thermal response. At the same time, optimal loadings and the resulting effects are sensitive to the type of nSiO_2_ used, whether fumed silica or silica aerogel, as well as to specific surface area, surface modification, dispersion method, and the vulcanization system. For reliable comparison of reported data, it is essential to clearly specify the type of nSiO_2_ and its specific surface area, filler content, the presence and type of silane-coupling agent, the mixing sequence, and the conditions of flame and ablative testing, together with the criteria used for calculating performance metrics.

### 2.3. Titanium Oxide Nanoparticles (nTiO_2_)

Titanium dioxide is widely used in polymer systems as a functional oxide additive capable of providing ultraviolet shielding and influencing thermo oxidative degradation processes. At the same time, the photocatalytic activity of titanium dioxide can enhance the photo-oxidative degradation of polymers if the particle surface is not passivated or if inhibitors are not employed. Therefore, the effect of titanium dioxide is conditionally beneficial and depends on formulation details and operating conditions [71,131,132]. In EPDM-based composite materials, titanium dioxide is usually not considered as an independent thermal insulation filler, but rather as a component of multicomponent additive packages, in combination with nSiO_2_ type fillers, CB, and other additives. In such systems, its contribution is intertwined with effects related to dispersion, rheology, and the structure of the degradation layer. Accordingly, correct interpretation requires comparable nTiO_2_-only and nTiO_2_-hybrid systems evaluated under identical test protocols. In several studies on EPDM composites containing complex inorganic additive packages that include titanium dioxide, an increase in thermal stability based on thermal analysis data is reported. The magnitude of the effect, for example, on the order of several tens of degrees, is specific to the particular formulation and additive combination employed [71,133,134,135].

In EPDM titanium dioxide compounds, rheological data, such as an increase in maximum torque, are interpreted as evidence of the reinforcing role of the dispersed titanium dioxide phase [132]. When assessing the influence of titanium dioxide, it is important to distinguish between changes in rheology or crosslinking behavior and changes in charring mechanisms. Conclusions regarding the enhanced charring or stabilization of the pyrolytic layer require direct evidence based on solid residue yield and char layer microstructure, as well as comparable evaluations of nTiO_2_ only and nTiO_2_ hybrid systems using the same test protocol. The morphology and uniformity of titanium dioxide dispersion, including agglomeration and local particle clustering, are critical factors and must be considered when interpreting both the mechanical and thermal properties [71,132].

For EPDM-based thermal protection composite materials, the char layer formed after flame exposure is commonly described as porous. In one study, it was explicitly reported that char porosity can exceed 60% [60]. The barrier function of the char layer is associated with its integrity and degree of densification, since a denser and more continuous layer can better resist erosion by combustion products and slow heat transfer [14,28].

Summary data on the influence of titanium dioxide on the thermogravimetric characteristics and charring behavior of EPDM at loadings in the range of 0–5 weight percent (wt%) are presented in Table 3. To enable a formulation-level interpretation, Table 3 summarizes the thermogravimetric parameters and residual mass for EPDM systems containing nTiO_2_ at 0–5 wt%. This compilation is intended to highlight the dose-dependent trends under comparable thermal analysis conditions, while separating nTiO_2_-only EPDM from multicomponent formulations where residue can be dominated by inorganic ash. At low loadings, on the order of 1 wt%, some studies report only minor changes in T_5_% and T_50_% compared with neat EPDM [136]. The compactness and integrity of the char layer play a decisive role in the ablative response of EPDM materials. A denser char layer can restrict the diffusion of oxygen-containing gases and reduce the contribution of convective and radiative heat transfer, which is associated with a decrease in T_back_ [14].

As indicated in Table 3, nTiO_2_ at low loadings typically only produces modest shifts in T_5_% and T_50_% relative to neat EPDM, whereas the residual mass increases with loading in the 1–5 wt% range. However, residue values reported as ash in air or for hybrid systems cannot be interpreted as polymer-derived char without additional evidence, such as residue composition analysis and microstructural characterization.

It has been shown that at higher TiO_2_ loadings in rubber matrices, the agglomeration of TiO_2_ particles can occur [137]. For EPDM-based composite materials containing additive packages that include TiO_2_, an increase in thermal stability has been reported based on combined TGA and Fourier transform infrared spectroscopy data [71]. In TiO_2_-filled polymer systems, rheological characteristics are commonly treated as a function of nanoparticle content and surface treatment, as demonstrated, for example, in isotactic polypropylene TiO_2_ systems [138].

Extended data on the influence of TiO_2_ in various EPDM formulations, including hybrid systems and composites containing other nanophases, are summarized in Table 4. For EPDM composite materials containing complex inorganic additive systems, such as TiO_2_ and graphene, TGA data indicate an increase in thermal stability on the order of 30–50 °C [71]. For EPDM-based thermal protection composite materials containing silica-type fillers, including fumed silica and silica aerogel, the formation of compact or denser char layers has been reported together with T_back_ values obtained in burn-through tests [28].

Mechanistic interpretations of the contribution of TiO_2_ to charring behavior and stabilization of the degradation layer should be formulated with caution. Reliable attribution requires direct comparative data for the TiO_2_ only and TiO_2_ hybrid systems evaluated under comparable test protocols. To avoid the over-attribution of residue or thermal shifts to TiO_2_ alone, Table 4 summarizes the representative EPDM formulations where TiO_2_ is either used as a standalone nanophase or is embedded in hybrid-additive packages. The table explicitly distinguishes polymer-derived residue from inorganic ash reported under oxidative conditions or in multicomponent systems.

Table 4 illustrates that large residue values in hybrid systems can be strongly affected by inorganic content and co-fillers, and therefore should not be interpreted as evidence of enhanced charring mechanisms without complementary microstructural and compositional validation. Consequently, claims regarding TiO_2_-driven char densification remain conditional and should be supported by side-by-side TiO_2_-only and hybrid comparisons conducted under identical protocols.

For EPDM-based composite materials, an improvement in ablative response is discussed in some studies in relation to changes in microstructure, including the state of the char layer [14], and to reduced heat transfer associated with a decrease in thermal conductivity [102]. In EPDM composites containing nanostructured additives, oxygen acetylene tests are reported with quantitative evaluation of LAR. Scanning electron microscopy analysis of the exposed surface and the post exposure residue is used to interpret the ablation mechanism [123]. For EPDM-based thermal protection composite materials containing silica type fillers, the formation of a more compact and denser char layer has been demonstrated, with reduced porosity confirmed by quantitative porosity parameters [28]. A conceptual illustration of how carbon black can improve char integrity and limit heat and O_2_ transport qualitatively follows a mechanism similar to that illustrated for oxide-filled systems in Figure 7.

In EPDM composite materials containing nTiO_2_, the influence of the filler depends on loading level, for example, 1, 3, and 5 wt% (Figure 6). At 5 wt%, the deterioration of certain mechanical metrics is attributed by the authors to agglomeration and non-uniform nanoparticle distribution [136].

In hybrid EPDM systems containing modified fumed silica, titanium dioxide, and graphene, an increase in thermal stability on the order of 30–50 °C is reported for a specific formulation produced by mechanical mixing [71]. For EPDM-based thermal protection composite materials containing silica systems such as fumed silica and silica aerogel, a denser char layer morphology, smaller pore size, and reduced porosity have been demonstrated. These features are associated with a decrease in T_back_ in burn-through tests [28]. Extrapolation of these effects to titanium dioxide modification should be formulated with caution and supported by direct and comparable experimental data.

### 2.4. Carbon Black (CB)

CB is widely used as an active filler in the rubber industry [139]. In studies devoted to surface modification of CB for rubber applications, filler–elastomer compatibility is discussed in terms of differences in surface energy, with EPDM frequently cited as an example of an elastomeric matrix [139]. CB is regarded as a carbon-based nanoparticle system and is described as grape-like aggregates composed of highly fused spherical particles [140]. For CB, features of surface chemistry are discussed, including oxygen-containing functional groups on the surface. The acceleration or modification of sulfur vulcanization and the possibility of forming interfacial bonds during vulcanization are also reported [32].

For EPDM filled with different grades of rubber-grade CB, experimental studies demonstrate differences in cure kinetics, evaluated by rheometric methods, and in mechanical properties, including tensile modulus at 300% elongation, tensile strength, and elongation at break [141]. CB is considered a key reinforcing filler in rubber materials, while it remains a matter of discussion whether interactions between polymer chains and the CB’s surface are predominantly due to physical adsorption or also involve the formation of chemical polymer–particle bonds [32].

For EPDM CB composites, it has been shown that differences in interactions with the surface of specific CB grades are reflected in deformation behavior. In particular, reductions in elongation at break are attributed to the EPDM molecules being firmly absorbed on the surface of N472 CB [141]. For EPDM vulcanizates filled with conducting CB, increasing CB loading results in higher hardness and tensile strength, with a tendency toward a plateau at loadings above approximately 40 phr. The elongation at break of unfilled vulcanizates exhibits a maximum at around 20 phr [142].

It has been shown that poor macrodispersion and the presence of micrometer-scale agglomerates are associated with reductions in tensile strength and elongation at break, as demonstrated for rubber systems, including styrene–butadiene rubber. In addition to reinforcement, CB can significantly alter the kinetics of sulfur vulcanization in elastomers. The reactive behavior of CB toward sulfur and commonly used vulcanization accelerators is also described in review studies [32].

For EPDM filled with CB, the parameters obtained from curing rheological measurements vary depending on the grade and characteristics of the filler. The minimum and maximum torque values, torque difference, scorch time, and the time required to reach optimal curing at elevated temperatures are all reported to be sensitive to the nature of CB. The minimum torque is commonly used as an indicator of polymer–filler interactions, while the optimum curing time is interpreted as a measure of curing efficiency [141]. In the melt rheology of EPDM, increasing the loading of conducting CB leads to changes in low shear and relative viscosity, as well as in yield stress. These effects are interpreted in terms of filler-structuring phenomena, including aggregation and network formation within the polymer matrix [143].

For EPDM ablative materials, it is emphasized that the char layer resists chemical erosion and mechanical removal, while the thermal stability and mechanical strength of the char layer are considered the key factors governing ablation performance [14]. For EPDM-based ablative materials, it has been shown that the strength and structure of the char layer are directly related to the ablation response. It is noted that the intrinsic char yield of the EPDM matrix is relatively low, and the resulting char layer may be weak and porous, which reduces resistance to erosion by gas flow and solid particles [60]. In one study, EPDM filled with CB exhibited a char yield of approximately 36.9–38.3% under inert thermogravimetric conditions [144]. For EPDM insulation materials used in SRMs, it is reported that the char yield of EPDM is relatively low and that the char layer is weak and porous and does not withstand erosion and scouring by hot gases and condensed particles [14].

For ceramifiable EPDM modifications, it is reported that the surface layer may undergo in situ transformation into a ceramic-like structure, which consolidates the carbon layer and enhances ablation resistance [60,145]. To quantify the grade-dependent reinforcement effect of rubber-grade CB in EPDM, representative mechanical properties (hardness, modulus at 300% elongation, tensile strength, elongation at break, and permanent set) for several CB grades are summarized in Table 5 [141].

As shown in Table 5, CB grade strongly affects stiffness and extensibility, indicating that the reinforcement level is not governed by loading alone but also by filler structure and polymer–filler interactions. In particular, high-modulus behavior combined with reduced elongation can be consistent with the stronger immobilization of EPDM chains on specific CB surfaces, which is frequently discussed for high-structure grades.

Hybridization in ablative composites is considered an effective approach for optimizing performance, including the ablation rate and erosion resistance [146]. For EPDM systems containing CB and graphene nanoplatelets, the material’s response is interpreted as a synergistic effect associated with the retardation of filler agglomeration. CB aggregates located on the surface of graphene nanoplatelets can bridge interparticle gaps and form additional conductive pathways [147]. In high-temperature particle-jet erosion studies of EPDM-based composites, the influence of carbon nanotube content and size has been demonstrated. Under conditions of a temperature of ~3100 °C, particle velocity of 42 m s^−1^, and particle concentration of 63.6 kg m^−3^, the reported charring rate decreased from 1.02 to 0.326 mm s^−1^ with appropriate nanotube incorporation [148]. In SRM simulations, it has been noted that EPDM formulations without silica or fiber reinforcement exhibit poor resistance and degradation under erosion regimes involving dense particle jets [5]. For EPDM insulation materials, compositional modification—for example, through the incorporation of multi-walled carbon nanotubes—has been shown to induce the evolution of the char layer’s morphology toward a dense porous structure in certain formulations [20].

In EPDM ablative materials, it is emphasized that the high thermal conductivity of ablative fillers can reduce thermal insulation efficiency and increase the risk of overheating the underlying matrix. To achieve a balance between ablation resistance and thermal insulation, a multilayer ablative and heat-management architecture has been proposed in which the interruption of the conductive pathways in the through thickness direction increases the effective heat transfer path while preserving the ablative performance of the ablative material layer [14].

To connect CB loading with ablation-relevant metrics, Table 6 compiles the representative torch and burn-through results reported for EPDM and CB systems, including MAR, LAR, and T_back_, at a fixed exposure time.

**Figure 12:**
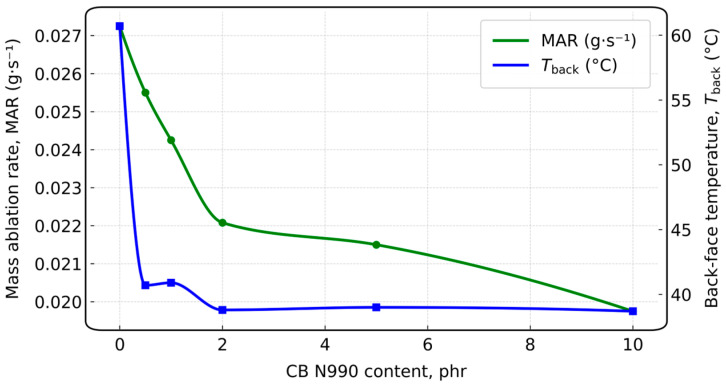
CB N990 dose–response in EPDM: MAR and T_back_ versus filler loading.

Within the N990 series, increasing CB content decreases MAR and reduces T_back_ relative to the neat reference under the same exposure duration, indicating improved surface retention and reduced mass loss. In contrast, highly conductive nanocarbon-rich formulations may exhibit very low LAR while showing elevated T_back_, highlighting the classical ablation-insulation trade-off associated with enhanced thermal conductivity. To provide a more detailed view of this dose-dependent behavior, the N990 dataset is summarized in Figure 12.

To generalize the above observations beyond individual test cases, Table 7 summarizes the main, repeatedly reported roles of CB and hybrid carbon-based systems in EPDM-based ablative composites, including reinforcement, aging sensitivity, and synergy in hybrid carbon networks.

Synergistic effects between carbon black and oxide nanoparticles can be rationalized by their complementary roles in stabilizing the evolving protective residue. Under controlled dispersion, carbon black forms a rubber–filler interphase with immobilized EPDM chain fragments, commonly described as bound rubber, together with adsorption-related junctions. These features enhance reinforcement efficiency and favor the formation of a more coherent carbonaceous precursor prior to charring [122,155]. In parallel, silica-type and other ceramic oxides promote the development of compact near-surface regions in the char, including silica-film-assisted glassy-skin features, which act as barriers limiting oxidizer access and slowing the thermo-oxidative consumption of the residue [59,156]

At elevated temperatures, silica-containing systems may additionally involve carbothermal reduction pathways between carbon and silica, leading to the redistribution of Si-containing phases and the modification of char yield and microstructure. These processes can alter erosion resistance and gas-transport pathways within the residue of EPDM-based insulators [20,157]. Overall, carbon-black–oxide hybrids can be interpreted as providing dual-scale stabilization of the char layer, in which carbon black primarily supports cohesion and retention of the carbonaceous skeleton, while oxide phases promote densification and near-surface sealing. The resulting ablative response depends on the carbon black-to-oxide ratio, since increased carbon black content can raise effective thermal conductivity, whereas strong pore-sealing and silica-related reactions can shift the dominant transport and residue-consumption pathways [20,158].

**Figure 13:**
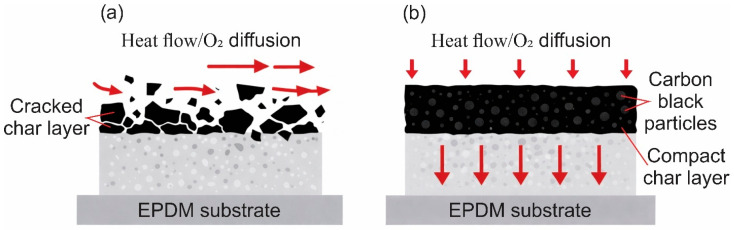
EPDM ablators with CB effect on char layer compactness and heat and O_2_ diffusion: (**a**) cracked char layer enabling enhanced heat and O_2_ diffusion to the EPDM substrate; (**b**) CB filled compact char layer with reduced heat and O_2_ diffusion.

Carbon phases contribute to reinforcement and char retention, while the highest erosion resistance is typically reported when the carbon phase is combined with oxide-type nanofillers that promote a denser and mechanically coherent protective framework. This concept is illustrated schematically in Figure 13.

Overall CB remains one of the most effective reinforcing and stabilizing fillers for EPDM composites. It enhances the strength and modulus, influences vulcanization kinetics, and increases the yield of carbonaceous residue during thermal degradation. However, the porous structure of the carbon residue and the limited ceramic character of the protective layer can reduce resistance to severe erosive damage under the most demanding operating conditions. As a result, the most pronounced thermal protection effects are typically achieved in hybrid systems, where the carbon phase provides reinforcement and char retention, while oxide nanoparticles promote the formation of a denser and more erosion resistant framework within the protective layer.

## 3. Comparative Analysis and Design Guidelines

The literature reviewed in this work indicates that the thermoablative performance of EPDM composites is governed less by the nominal presence of a high-temperature filler and more by how the filler modifies the coupled sequence of (1) vulcanization network formation, (2) thermal decomposition and residue generation, and (3) the structure, permeability, and mechanical stability of the evolving char layer in a high-enthalpy, oxidizing, gas-dynamic environment. The practical limitation of EPDM ablators is repeatedly linked to insufficient integrity and barrier properties of the char layer, its oxidation, and flow-induced mechanical removal, while the engineering transfer of published results is further complicated by differences in test conditions and reporting formats.

A mechanistic reading of the datasets supports a functional classification of 0D nanofillers into a small number of dominant roles: (1) reinforcement and char retention; (2) barrier structuring and permeability control in the degradation layer; (3) thermo-oxidative stabilization and residue promotion; and (4) regulation of vulcanization kinetics and network density. This classification is not purely academic. It directly maps to the principal failure modes seen in EPDM ablators, including brittle, porous char leading to spallation and erosion, oxygen access through connected pores accelerating oxidative burnout, excessive back-face heating due to insufficiently insulating degradation architecture, and formulation sensitivity where small changes in cure state or dispersion shift the char from cohesive to fragmenting.

CB illustrates the classical strength–insulation compromise in ablatives. In the torch or burn-through datasets compiled in this review, increasing CB content in a comparable series reduces mass loss and lowers T_back_ relative to neat EPDM under identical exposure duration, indicating improved surface retention and reduced material removal. At the same time, highly conductive nanocarbon-rich formulations can exhibit very low LAR while producing elevated T_back_, explicitly demonstrating that erosion resistance can be gained at the expense of thermal insulation because conductive pathways intensify through thickness heat transfer.

This trade-off implies that the optimal CB loading depends on the governing design constraint. If the limiting condition is mechanical washout and surface recession, carbon phases are beneficial. If the limiting condition is backside overheating, conductive percolation must be controlled. This can be achieved through architectural concepts that interrupt conductive pathways in the thickness direction while preserving a robust ablative surface layer.

nSiO_2_ is often reported for thermal protection behavior because it simultaneously improves mechanical stability and promotes the formation of a more stable and denser char, which is commonly associated with reduced T_back_ and improved resistance to burn-through. However, this predictability is conditional on interfacial design and dispersion quality. Reported limitations at elevated silica loadings include agglomeration and restricted chain mobility, and in some systems, silica is shown to influence the effective crosslink density through interactions with the crosslinking system and coupling agents. Accordingly, silica-containing EPDM requires explicit specification of the nSiO_2_ type, specific surface area, coupling chemistry, mixing sequence, and testing protocol to make comparisons meaningful.

The practical relevance is clear because the char formed after flame exposure is often highly porous, with reported porosity values exceeding 60%, so any filler that densifies the char layer and reduces connected pore pathways can shift the governing mechanism from oxygen-enabled burnout to barrier-controlled recession.

Titanium dioxide occupies a more ambiguous position. It is widely used as a functional oxide capable of UV shielding and influencing thermo-oxidative degradation, but its photocatalytic activity can enhance photo-oxidative degradation if surfaces are not passivated or inhibitors are not used, making the net effect formulation- and condition-dependent. In EPDM composites, TiO_2_ is frequently embedded in multicomponent additive packages, and correct interpretation therefore requires side-by-side TiO_2_-only and TiO_2_ hybrid systems evaluated under identical protocols.

Where comparable thermal analysis data exist, nTiO_2_ in the 1–5 wt% range shows a dose-dependent increase in residual mass, while shifts in T_5_% and T_50_% can be modest at low loadings. Critically, residue reported as ash in oxidative conditions or in hybrid systems cannot be equated with polymer-derived char without compositional validation.

From a design standpoint, TiO_2_ can be viewed as a supportive stabilizer and residue promoter, with its use appearing most rational in hybrid architectures rather than as a standalone thermal insulation nanofiller.

Zinc oxide remains primarily a vulcanization-active component rather than a direct thermal insulation additive. The reviewed EPDM studies emphasize that ZnO and nZnO influence crosslinking kinetics, network density, and cure reproducibility, and that replacement of conventional ZnO with nZnO can change degradation kinetic parameters and increase crosslink density, which indirectly affects the ability of the material to preserve integrity during heating and to form a stable protective layer.

At the same time, direct quantitative comparisons of ZnO versus nZnO contributions to LAR and MAR under high heat flux remain limited, so claims of ablation reduction by nZnO should be framed as conditional and network-mediated rather than guaranteed.

Taken together, the reviewed studies indicate that hybrid designs are frequently reported to improve performance by combining carbon-driven char cohesion and retention with oxide-assisted barrier formation and densification. Carbon phases contribute to reinforcement and char retention, while oxide nanoparticles improve barrier character, densification, and resistance to oxidative damage. This interpretation is consistent with reports that carbon-rich residues can remain porous and mechanically vulnerable under severe erosive loading, whereas oxide-assisted surface enrichment and densification can improve barrier integrity in the near-surface region.

The engineering implication of Table 8 and Table 9 is that single-filler optimization should be treated as a constrained special case. Most EPDM ablator failure modes are multi-causal (erosion plus oxidation plus backside heating), so a functional split between reinforcement and barrier formation is typically required to avoid over-optimizing one metric while degrading another, especially under the ablation-insulation trade-off seen in conductive carbon-rich systems.

From a design standpoint, Table 8 and Table 9 is intended as a decision-support guide for formulation screening. First, identify the dominant limiting mode in the target environment, whether recession is driven by mechanical removal, burnout is driven by oxidation through connected pores, or overheating is driven by insufficient thermal resistance. Second, select a primary functional filler to address that mode, using carbon for cohesion and retention, silica-type fillers for barrier formation and densification, and cure-active ZnO for network reproducibility. Third, add a complementary phase if the operating regime is mixed, which is the most common case in SRM-relevant exposure. This hybrid-first logic follows directly from the review-level conclusion that the maximum effect is generally achieved when carbon improves char cohesion while oxides enhance barrier performance and oxidation resistance.

Finally, the comparability problem must be treated as part of the design guideline, not as an editorial note. Because LAR, MAR, T_back_, and residue yield are often measured under different geometries and regimes, the most defensible comparisons are within-study and within-protocol, while cross-study ranking should rely on mechanism-consistent trends rather than absolute values. Accordingly, transparent reporting improves comparability and engineering transfer, particularly when filler type and surface area, coupling chemistry, mixing sequence, cure system, and the ablation or flame-test configuration are reported together with consistent definitions of LAR, MAR, T_back_, and residue yield.

## 4. Conclusions

This review shows that 0D nanofillers are effective tools for the targeted modification of EPDM composites intended for thermoablative service because their contribution is expressed not as an abstract increase in thermal stability, but rather through the control of a coupled chain of processes, including vulcanization kinetics and crosslink density, pyrolysis and residue formation, and the structure, permeability, and mechanical integrity of the evolving char layer in an oxidizing gas dynamic environment.

From an engineering standpoint, nanofiller selection is typically guided by the dominant degradation mechanism and the imposed thermal loading regime, rather than by the notion of a universally optimal filler. nSiO_2_ primarily acts through barrier structuring of the degradation layer and improved char integrity, while the magnitude of the effect is critically governed by dispersion quality and interfacial design. Titanium dioxide can contribute to thermo oxidative stabilization and higher residue levels, yet its most rational use is within multicomponent formulations, where it functions as part of a coordinated additive package. nZnO remains mainly a regulator of vulcanization and network reproducibility. Therefore, its influence on ablation-related losses should be treated as indirect and contingent on formulation details and the experimental configuration.

Overall, a frequently supported architecture is a hybrid approach in which a carbon phase promotes reinforcement and char retention, while an oxide phase enhances the barrier functionality and structural stability of the degradation layer, improving resistance to oxidation and flow induced removal. At the same time, interpretation of the literature is constrained by differences in test protocols and reporting formats. Progress in the field therefore requires improved comparability and a broader base of direct ablation measurements for 0D nanofillers under standardized conditions, with unambiguous reporting of composition, processing and mixing routes, and consistent definitions for LAR, MAR, T_back_, and residue yield.

## Figures and Tables

**Table 1:** Mechanical performance of EPDM composites modified with nSiO_2_: comparative tensile properties and hardness across formulations and hybrid systems.

Filler	Filler Size, nm	Treatment	Dispersion	EPDM and Filler (phr)	Tensile Strength, MPa	Elongation at Break, %	Elastic Modulus, MPa	Hardness, Shore A	Ref.
-	-	-	-	100/0	1.11	244.5	-	48.4	[116]
nSiO_2_	12	-	Uniform	100/6	2.04	220.2	-	58.1	[116]
micro-SiO_2_	5000	-	Uniform	100/23	1.90	223.4	-	57.3	[116]
micro-SiO_2_ + nSiO_2_	5000 + 12	-	Uniform	100/23 + 6	2.46	196.3	-	68.3	[116]
polyimide	74,000	-	-	100/5	2.52	345.0	2.20	55.0	[117]
polyimide + nSiO_2_	74,000 + 5–15	A-189	Uniform	100/5 + 5	4.08	334.1	3.65	58.0	[117]
nSiO_2_	50–80	SGPE	Uniform	50/2.5	3.88	637.00	1.20 (M100)	-	[118]
nSiO_2_	50–80	SGPE	Uniform	50/5.0	4.43	679.00	2.14 (M100)	-	[118]
nSiO_2_	50–80	SGPE	Uniform	50/7.5	4.45	701.00	1.85 (M100)	-	[118]
nSiO_2_	50–80	SGPE	Non-uniform	50/10.0	3.52	712.00	2.48 (M100)	-	[118]
Kevlar fiber	-	-	Uniform	100/10	3.93	228	51.65	80	[119]
Kevlar fiber + nSiO_2_	12–16	SCA	Uniform	100/10 + 3	4.88	376	64.09	93	[119]
nSiO_2_	~100	-	Non-uniform	100/11.1	4.9 ± 2.1	2863 ± 101	2.2 ± 0.2	-	[120]
nSiO_2_ + specific filler	~100	TESPT	Non-uniform	100/11.1	10.0 ± 1.6	1570 ± 14	3.0 ± 0.0	-	[120]
nSiO_2_	~100	-	Non-uniform	100/25.0	3.4 ± 0.3	1865 ± 35	3.2 ± 0.2	-	[120]
nSiO_2_ + specific filler	~100	TESPT	Uniform	100/25.0	4.4 ± 2.1	963 ± 10	4.8 ± 0.9	-	[120]

Note: All averaged data are presented as mean values; the uncertainty (±) corresponds to typical variations reported in the cited sources. SCA and A-189: silane coupling agent; TESPT: tetrasulfide silane; SGPE: Silane-grafted polyethylene; M100: modulus at 100% elongation.

**Table 2:** Ablation performance of EPDM composites under different gas velocities: LAR and MAR and qualitative char integrity for silica- and fiber-modified systems.

Filler	Filler Content, phr	LAR, mm·s^−1^	MAR, g·s^−1^·mm^−2^	MAR, g·s^−1^	Char and Features	Ref.
Fumed silica	20	0.117	125.3	-	More continuous, cohesive char	[5]
Silica	0	0.156	149.9	-	More porous and rough char	[5]
Fumed silica	20	0.163	233.1	-	Cracks and partial downstream delamination	[5]
Silica	0	0.374	319.9	-	Pronounced peeling and easily erodible char	[5]
Silica + aramid fiber	20 + 15	0.163	233.1	-	Cracks, partial downstream delamination	[5]
Silica + aramid fiber	20 + 0	0.296	263.9	-	Higher tendency to peeling, char stripping	[5]
CNT + nSiO_2_	10 + 0	~0.027	-	~0.038	Irregular residue with peeling traces and moderate pits	[123]
CNT + nSiO_2_	10 + nSiO_2_ shell	~0.0130	-	~0.0335	Smoother surface with shallow pits and more uniform residue	[123]

**Table 3:** Thermogravimetric decomposition parameters of EPDM composites containing titanium dioxide: onset and mid-point degradation temperatures and residue formation trends.

Filler	Filler Size, nm	Dispersion	Content	T_5_% (°C)	T_50_% (°C)	Char Yield, Features	Ref.
Neat EPDM	-	-	0 phr	343	420	Ash ~0.3 wt%	[71]
nTiO_2_	100	-	0 wt%	410.47	463.60	Pyrolysis residue ~2 wt%	[136]
nTiO_2_	100	-	1 wt%	430.21	467.51	Pyrolysis residue ~5 wt%	[136]
nTiO_2_	100	-	3 wt%	436.84	469.65	Pyrolysis residue ~10 wt%	[136]
nTiO_2_	100	Non-uniform	5 wt%	438.36	470.32	Pyrolysis residue ~13 wt%	[136]
nTiO_2_	28–34	Uniform	20 phr	350	448	Ash ~17 wt%	[71]
Silicone rubber + micro-TiO_2_	28–34	Phase separation	50 phr + 20 phr	303	503	Ash ~34 wt%	[71]

**Table 4:** Thermal stability and residue formation of TiO_2_ based additive systems in EPDM and EPDM silicone rubber blends: comparative degradation temperatures and ash dominated residue levels.

Filler	Filler Content, phr	Thermal Parameter, °C	Residue, wt%	Ref.
TiO_2_	20	350	17	[71]
TiO_2_ + graphene	20 + 2	353	18.1	[71]
Silicon rubber + TiO_2_ + graphene	50 + 20 + 2	331	32.3	[71]

**Table 5:** Mechanical properties of EPDM compounds filled with different rubber-grade carbon-black types: hardness, modulus at 300% elongation, tensile strength, elongation at break, and permanent set.

Filler	Filler Size, nm	Dispersion	EPDM and Filler (phr)	Hardness, Shore A	Modulus at 300% Elongation, MPa	Tensile Strength, MPa	Elongation at Break,%	Permanent Set,%
SCB	200–500	Uniform, aggregates observed	100/30	57	4.67	8.44	510	12
N770	180–300	Uniform, aggregates larger	100/30	52	2.91	12.12	640	17
N550	40–60	Uniform, aggregates with pores	100/30	60	5.17	15.04	526	15
N330	25–35	Uniform, fine aggregates	100/30	59	5.23	18.38	571	15
N472	15–20	Uniform	100/30	79	15.12	17.76	345	17

Note: SCB, N770, N550, N330, and N472 are CB filler grades used as reinforcing fillers in EPDM and rubber compounds.

**Table 6:** Ablation response of EPDM composites filled with CB: MAR, LAR, and T_back_ at specified exposure times.

Filler	Filler Size, nm	Dispersion	Filler Content, phr	LAR (mm·s^−1^)	MAR (g·s^−1^)	T_back_, °C	Key Features	Ref.
CB N990	280	-	0 (neat EPDM)	-	0.02725	60.7 after 40 s	Baseline behavior with pronounced back-face heating	[149]
CB N990	280	-	10	-	0.01975	38.7 after 40 s	Minimum mass loss of 6.39% within the studied series	[149]
CB N220	20–25	Uniform	20	0.011	-	160 after 60 s	Very low LAR accompanied by elevated T_back_ due to enhanced thermal conductivity	[150]
CB hybrid system	20–25	Uniform	10	0.013	-	87 after 60 s	Balanced performance combining low LAR and moderate T_back_	[150]
CB hybrid system	20–25	Uniform	5	0.012	-	70 after 60 s	Lowest T_back_ among the CB based systems in this dataset	[150]

**Table 7:** Summary of reported roles of CB and hybrid carbon nanophases in EPDM-based ablative composites.

Filler	Filler Content (phr)	Main Effects	Ref.
CB	20–50	Increased crosslink density and vulcanization rate leading to higher modulus and tensile strength and reduced swelling due to enhanced filler-rubber interactions and effective network formation	[32,141,151]
		Under thermo-oxidative aging mechanical properties degrade while CB loading strongly affects degradation kinetics and retention of service properties	[152,153]
CB + Graphene Nanoplatelets hybrid system	20–50 + up to ~7	Formation of a hybrid carbon network reduces aggregation and improves reinforcement efficiency resulting in enhanced thermophysical and barrier mechanical properties at optimized filler ratios	[147,154]

**Table 8:** Comparative characteristics of 0D nanofillers by their dominant functional roles in EPDM ablators.

Filler	Primary Role and Function
CB	CB Reinforces EPDM and strengthens char, less flow damage
nSiO_2_	nSiO_2_ Densifies char and blocks oxygen pathways
Silica aerogel	Silica aerogel Low heat transfer, char effect depends on residue structure
nTiO_2_	nTiO_2_ Conditional stabilization and residue support, best in hybrids
nZnO	nZnO Cure control for stable crosslinking and repeatable ablation
Hybrid carbon + oxide (e.g., CB + nSiO_2_ or CB + TiO_2_-type)	CB + nSiO_2_ or CB + nTiO_2_ Carbon for strength, oxide for barrier, best combined stability

**Table 9:** Engineering-oriented overview of 0D nanofillers for EPDM ablative composites: mechanisms, degradation scenarios, and design principles.

Engineering Issue	Key Mechanism	Suitable 0D Nanofiller	Expected Effect	Main Limitation or Risks
Char cracking and removal by gas flow	Make the char stronger and more cohesive	CB, CB + nTiO_2_, CB + nSiO_2_	More continuous char, less material loss, sometimes lower ablation rates	Can increase T_back_
Porous char letting oxygen in	Build a denser barrier with lower permeability	nSiO_2_, CB + nSiO_2_	Less oxygen access, slower oxidation, char lasts longer	Works only with good dispersion and interface
Fast oxidation of char at high temperature	Densify the residue and support a protective layer	nSiO_2_, nTiO_2_ in mixed systems	Better oxidation resistance, lower mass loss	Residue can become stiff and crack
High T_back_	Reduce heat transfer through the material	nSiO_2_, porous nSiO_2_ concepts	Slower heating of the back face	Too much densification can increase heat transfer
Poor batch reproducibility	Stabilize curing and crosslink density	nZnO as curing regulator	More consistent network and more stable ablative response	Indirect effect and strongly formulation dependent
Need both erosion resistance and insulation	Combine reinforcement and barrier formation	CB + nSiO_2_, CB + nTiO_2_, multilayer concepts	Lower surface loss and controlled T_back_	More complex formulations and higher risk of negative interactions

## Data Availability

No new data were created or analyzed in this study. Data sharing is not applicable to this article.

## Abbreviations

The following abbreviations are used in this manuscript:

SRM	Solid Rocket Motor
EPDM	Ethylene Propylene Diene Monomer
LAR	Linear Ablation Rate
MAR	Mass Ablation Rate
T_back_	Back-Face Temperature
TGA	Thermogravimetric Analysis
wt%	Weight Percent
phr	Parts Per Hundred Rubber
T_5_%	Temperature At 5% Mass Loss TGA
M300	Modulus At 300% Elongation
nSiO_2_	Silicon dioxide nanoparticles
nZnO	Zinc oxide nanoparticles
nTiO_2_	Titanium dioxide nanoparticles
